# Synthesis of Si-Sb-ZnO Composites as High-Performance Anodes for Lithium-ion Batteries

**DOI:** 10.1186/s11671-015-1128-4

**Published:** 2015-10-23

**Authors:** Yongliang Li, Liang Huang, Peixin Zhang, Xiangzhong Ren, Libo Deng

**Affiliations:** School of Chemistry and Environmental Engineering, Shenzhen University, Shenzhen, Guangdong 518060 People’s Republic of China

**Keywords:** Lithium-ion batteries, Si-Sb-ZnO composite materials, Electrochemical properties

## Abstract

The Si-Sb-ZnO composites were prepared by a chemical reduction-mechanical alloying method and were employed as anode materials for lithium-ion batteries. The electrochemical performance of the Si-Sb alloy was significantly improved by the addition of ZnO nanoparticles. Especially, the initial specific charge and discharge capacities for Si-Sb-(ZnO)_0.3_ composite were 845.1 and 1301.5 mAh/g, respectively, while the initial coulombic efficiency was 64.9 %. The capacity remained at 690 mAh/g after 200 cycles, and the capacity retention ratio was 81.6 %, which demonstrated excellent cycling stability and rate capability of the composite materials.

**Electronic supplementary material:**

The online version of this article (doi:10.1186/s11671-015-1128-4) contains supplementary material, which is available to authorized users.

## Background

Lithium-ion batteries are promising energy storage systems for the rapid growing field of hybrid electric vehicles and electric vehicles due to their high energy density and long cycle life, etc [1–3]. Silicon (Si) is an ideal anode material for lithium-ion battery owing to its ultrahigh specific capacity of ~4200 mAh/g, low discharging potential, and safety. However, poor electrical conductivity and the huge volume change during lithium-ion insertion/extraction processes are two critical obstacles for Si-based anode materials [4, 5].

Recently, Si-based alloys have been developed to overcome the two disadvantages; the Si-M (M = Mg [6, 7], Sn [8, 9], Ag [10, 11], Zn [12, 13], Fe [14, 15], and Ni [16, 17]) alloys are able to alleviate the volume expansion and consequently increase the cyclability for the batteries. In our previous research, the immiscible Si-Sb alloy was synthesized and demonstrated moderate electrochemical and cycling performance with a reversible capacity of 596.4 mAh/g [18]. Nevertheless, the Si-based alloy particles lose electrical contact with each other because of the huge volume expansion during charge/discharge processes. Therefore, improving the cycling stability as well as maintaining the high capacity has been an attractive research area in Si-based anode materials.

Zinc oxide (ZnO) was one of the candidates for anode materials due to its high theoretical capacity, low cost, and nature abundance [19]. However, its poor cyclability, caused by volume expansion during the charge/discharge, affects the practical application [20–22]. In this work, ZnO was introduced to the binary Si-Sb materials by a chemical reduction-mechanical alloying method. ZnO has a high theoretical capacity of 978 mAh/g, which could improve the specific capacity of the composite materials. In addition, ZnO was able to effectively relieve the agglomeration of the Si-Sb alloy particles, preventing the loss of electric contact between the current collector and electrode materials induced by the volume expansion. The electrochemical performance of Si-Sb-(ZnO)_*x*_ (*x* = 0, 0.1, 0.3, 0.5, 0.7, 0.9) composite anodes was studied, and the dependence of the cyclic behavior of the electrodes was discussed. It was found that when the molar ratio of ZnO was 0.3, the initial specific charge capacity and specific discharge capacity of the synthesized Si-Sb-(ZnO)_0.3_ composite anode material were 870.3 and 1301.5 mAh/g, respectively. The addition of ZnO was able to effectively relieve the agglomeration of the Si-Sb alloy particles, resulting in greatly improved cycling stability for the Si-Sb-(ZnO)_*x*_ composite anode materials.

## Methods

### Synthesis of Materials

All reagents were of analytical quality and used without any further purification. Si-Sb-(ZnO)_*x*_ composite materials were synthesized by a chemical reduction-mechanical alloying method. Typically, 0.1 mol of SbCl_3_, C_6_H_5_Na_3_O_7_, and Zn(NO_3_)_2_ with the molar ratio of 1:25:*x* (*x* = 0, 0.1, 0.3, 0.5, 0.7, 0.9) as well as a 0.2 mol aqueous solution of NaBH_4_ (pH > 12) were prepared, respectively. Excess amount of NaBH_4_ was added dropwise to the SbCl_3_/Zn(NO_3_)_2_ solution with strong magnetic stirring at room temperature. The solutions were aged in a water bath at 80 °C for 5 h. The precipitates were collected by centrifugation, washed with distilled water, and then mixed with 0.1 mol of Si nanopowders for the ball-milling process. The milling was performed under argon atmosphere for 15 h at 500 rpm, and the final products were dried at 120 °C for 10 h under vacuum and then stored in the Ar-filled glove box.

### Physical Characterizations

The phases of the composite materials were analyzed using a D8 Advance X-ray diffraction (XRD) instrument. The microstructure and morphology of the particles were observed using field-emission scanning electron microscopy (FE-SEM) by a Hitachi Limited S-3400 N instrument.

### Electrochemical Characterizations

To evaluate their electrochemical properties, 70 wt.% of active materials, 15 wt.% of acetylene black and 15 wt.% of carboxyl methyl cellulose (CMC), and styrene butadiene rubber (SBR) were mixed to form slurry, which was then pressed onto a copper grid as electrodes and dried at 120 °C for 10 h under vacuum. The loading of active materials was about 1.2 mg cm^−2^ on the electrodes.

The electrochemical performances were measured using coin cells assembled in an argon-filled glove box; these cells contained metallic lithium foil as a counter electrode, 1 M LiPF_6_ in ethylene carbonate (EC)-dimethyl carbonate (DMC) (1:1 v/v) as the electrolyte, Celgard 2400 membrane as a separator, and the synthesized composite alloy as a working electrode. The galvanostatic charge/discharge performance was assessed using a CT2001A battery tester from 0.1 to 2.0 V (vs. Li^+^/Li) at a constant current density of 0.1 mA/cm^2^ at room temperature unless noted. Cyclic voltammetry (CV) from 0 to 2.0 V (vs. Li/Li^+^) at 0.01 mV/s and electrochemical impedance spectroscopy (EIS) in the frequency range 10^5^ to 0.01 Hz and with an amplitude of 5 mV were performed using a Solartron 1470e electrochemical testing system.

## Results and Discussion

**Fig. 1:**
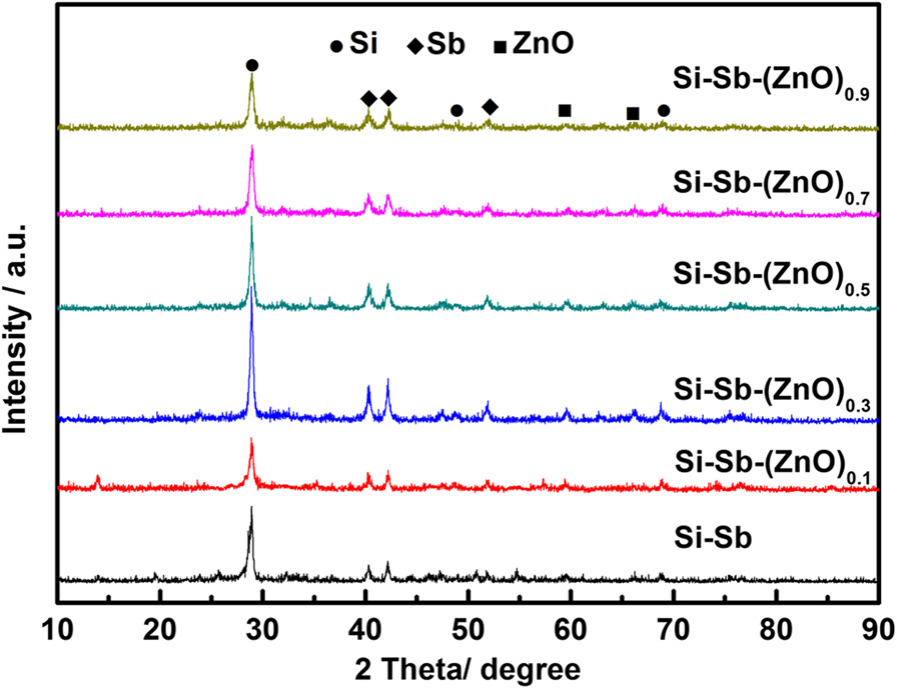
XRD patterns of the Si-Sb-(ZnO)_*x*_ composite materials

**Fig. 2:**
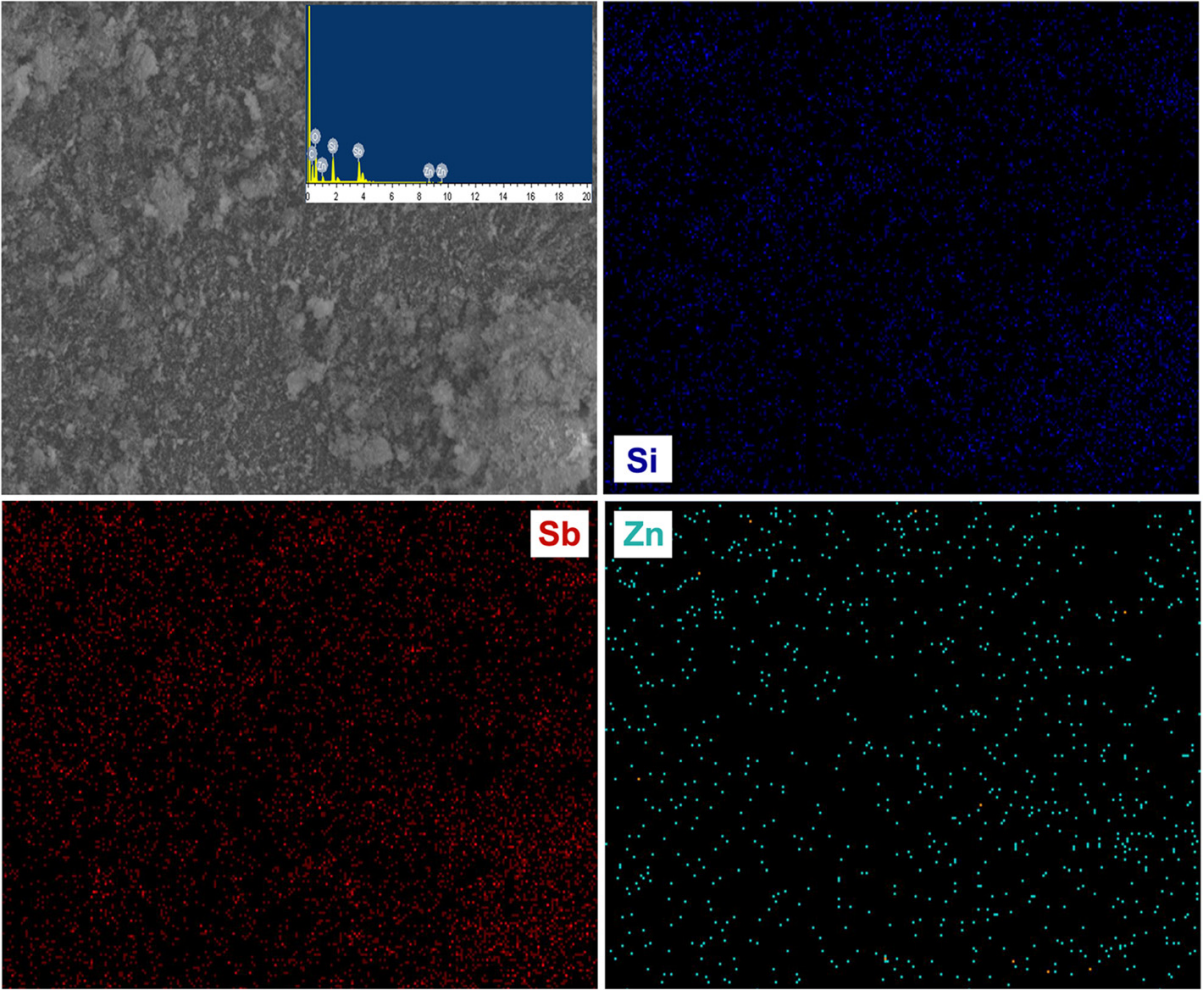
SEM and the correlated elements mapping images for Si-Sb-(ZnO)_0.3_ anode material

Figure 1 shows the XRD patterns of Si-Sb-(ZnO)_*x*_ composites obtained by chemical reduction-mechanical alloying method. The diffraction patterns of the composites consist of Si, Sb, and ZnO peaks, indicating that there is no intermetallic compound formed in the synthesis process. The characteristic peak intensities of Si and Sb are gradually increased with the ZnO content increased from 0 to 30 wt.%, and the peak intensities decreased when the ZnO content continued to increase. The results indicate that the Si-Sb-(ZnO)_0.3_ composite possessed the best crystallinity. The back-scattered image of Si-Sb-(ZnO)_0.3_ composite presented in Fig. 2 shows that Si, Sb, and ZnO are integrated without an apparent distinction in phases. According to the EDX analysis and the element mapping, the composite is consisted of Si (10.13 at.%), Sb (10.63 at.%), and Zn (2.91 at.%) in the scanning area, which are close to theoretical values (Table 1).

**Table 1:** The quantification results of the EDX measurements for Si-Sb-(ZnO)_0.3_ anode material

Element	wt.%	at.%
C K	19.04	35.72
O K	25.17	40.31
Si K	8.91	10.43
Zn L	5.89	2.91
Sb L	40.99	10.63
Total	100.00	

**Fig. 3:**
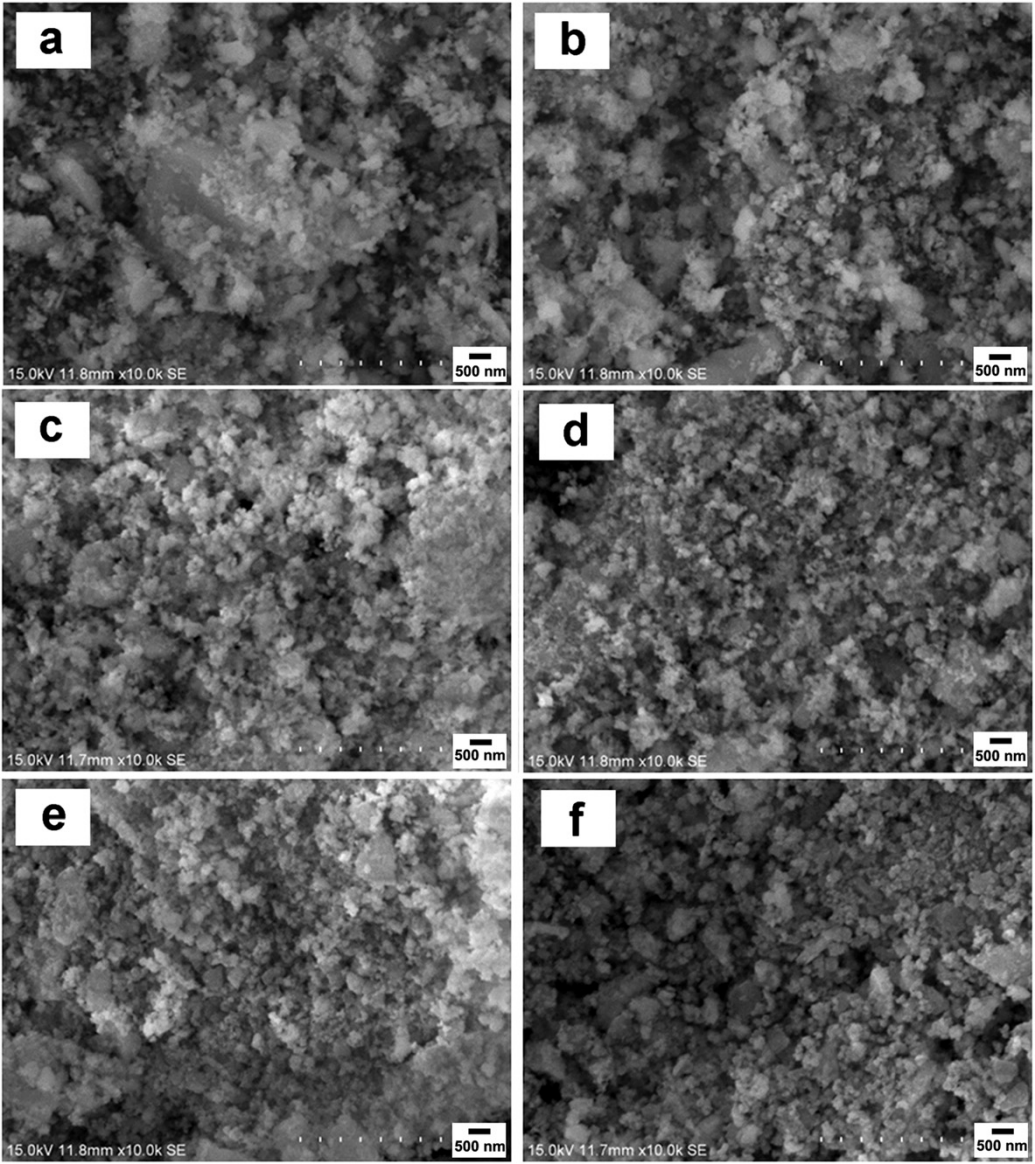
SEM images of Si-Sb-(ZnO)_*x*_ composite materials with different *x*: 0 (**a**), 0.1 (**b**), 0.3 (**c**), 0.5 (**d**), 0.7 (**e**), 0.9 (**f**)

Figure 3 shows the SEM images of Si-Sb-(ZnO)_*x*_ (*x* = 0, 0.1, 0.3, 0.5, 0.7, 0.9) composite materials. As shown in Fig. 3a, the Si-Sb alloy particles are with irregular morphology and some of them are aggregated to form big particles. With the addition of ZnO, the distribution of the composite becomes uniform, indicating that ZnO plays a role in preventing the reunion, reducing the mechanical stress between the particles. The Si-Sb-(ZnO)_*x*_ samples were composed of tiny particles which formed homogenous secondary particles; the particle size of the nano-composite materials was about 100 nm.

**Fig. 4:**
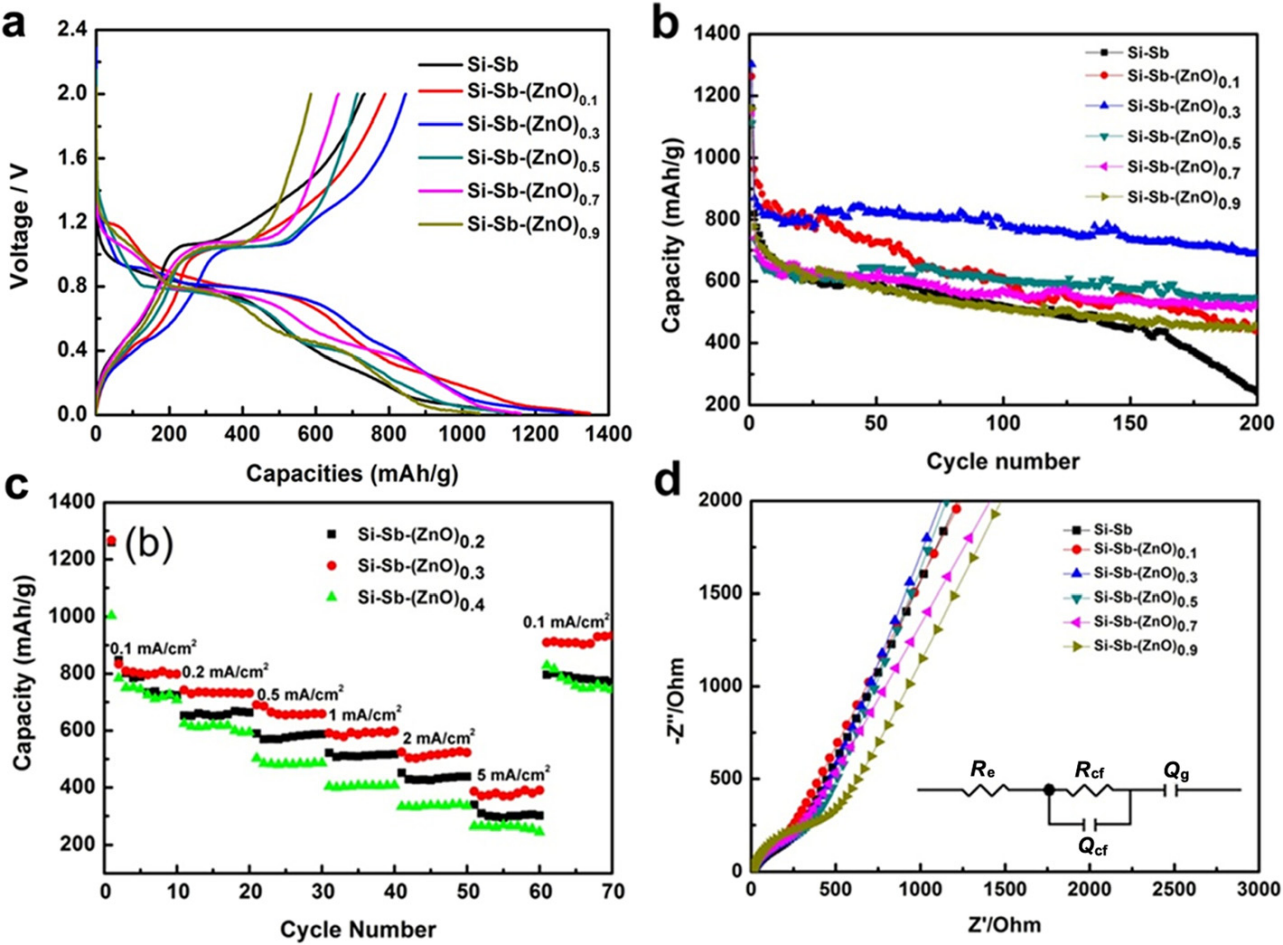
Voltage profiles (**a**), cycling performances (**b**), rate capabilities (**c**), and electrochemical impedance spectra of Si-Sb and Si-Sb-(ZnO)_*x*_ composite materials (**d**). The *inset* of **d** is the associated equivalent circuit

Figure 4a shows the curves of initial discharge and charge profiles of Si-Sb-(ZnO)_*x*_ (*x* = 0, 0.1, 0.3, 0.5, 0.7, 0.9) composite anodes at 0.1 mA/cm^2^. The similar trends of all the curves indicate that addition of ZnO hardly affects the mechanism of lithium extraction and insertion for composites. The initial discharge curves are divided into five parts. The first part shows a rapid decrease over 0.8 V, which corresponds to irreversible reaction such as the electrolyte decomposition and the formation of solid electrolyte interface (SEI) film. A discharge plateau appears at about 0.8 V, which is likely due to the reaction of Si-Sb with lithium. The reaction is: 3Li + Si-Sb → Li_3_Sb + Si. The third part of the curve is from 0.8 to 0.5 V, which corresponds to the formation of various Li_*x*_Sb compounds. Another discharge plateau appears at about 0.5–0.2 V, which is contributed from the reaction of ZnO with lithium, resulting in the formation of Li_2_Zn_5_ (0.64 V), LiZn_2_ (0.5 V), Li_2_Zn_3_ (0.24 V), and LiZn (0.18 V), etc [23]. With the ZnO content increased, this plateau was extended. The fifth part of the curve is below 0.2 V, corresponding to the lithium reaction with Si to form Li_*x*_Si compounds [24, 25]. It is noted that the lithium intercalation potentials for the contents in Si-Sb-(ZnO)_*x*_ composite materials are different; they can serve as matrices for each other to buffer the volume expansion during the lithium intercalation process. Therefore, when Sb reacts with Li at about 0.8 V, Si and ZnO would buffer the volume expansion for Sb; when discharged to about 0.5 V, Si and Sb would alleviate the volume effect of ZnO; Sb and ZnO would inhibit the volume change for Si when the voltage decrease to about 0.2 V. Such processes can improve the electrochemical properties of the composite materials.

Cycling performances of Si-Sb-(ZnO)_*x*_ composite anode materials were investigated by galvanostatic charge/discharge at 0.1 mA/cm^2^; the results are shown in Fig. 4b. The initial discharge capacities of Si-Sb-(ZnO)_*x*_ (*x* = 0, 0.1, 0.3, 0.5, 0.7, 0.9) composites are 1161.2, 1262.9, 1301.5, 1159, 1146.9, and 1112.2 mAh/g, respectively. Due to the formation of Sb and Si oxides during the synthesis process which would react with lithium in the first discharge cycle, the irreversible capacities are also increased. The discharge capacities for the second cycle are 818.5, 963, 870.3, 734.4, 740.3, and 777 mAh/g, and the capacities retained at 242.4, 436.8, 690.6, 548.1, 519, and 456.7 mAh/g after 200 cycles. The Si-Sb-(ZnO)_0.3_ exhibited 2.5 times of capacity retention compared to Si-Sb alloy and showed the best cycling performance among the composite anode materials. It was demonstrated that with the addition of ZnO, the electrochemical performance of Si-Sb-(ZnO)_*x*_ was significantly improved (Additional file 1: Figure S1). The reason is that ZnO has a high theoretical capacity of 978 mAh/g, which could improve the specific capacity of the composite materials. In addition, ZnO was able to effectively relieve the agglomeration of the Si-Sb alloy particles, preventing the loss of electric contact between the current collector and electrode materials induced by volume expansion.

To confirm the superior electrochemical properties of the Si-Sb-(ZnO)_0.3_ composite material, the performance at different current densities was evaluated. As shown in Fig. 4c, the anodes discharged at increasing current densities during the preceding 60 cycles. The initial discharge capacities of Si-Sb-(ZnO)_*x*_ (*x* = 0.2, 0.3, 0.4) are 1260, 1267.3 and 1004 mAh/g, respectively, and the average specific capacities decreased as the current density increased. The anode materials became fully lithiated at a low current density, therefore exhibiting high capacities; while at high current densities, the diffusion of lithium ions in the liquid phase and solid electrode are different, resulting in different equilibrium rates for the reactions, while the capacities decreased. The Si-Sb-(ZnO)_0.3_ composite anode material exhibited the highest discharge capacities among the three composites, even at high current density of 5 mA/cm^2^. When the current density varied from 5 to 0.1 mA/cm^2^, the discharge capacity of Si-Sb-(ZnO)_0.3_ recovered to 910.8 mAh/g, showing excellent rate capability.

Figure 4d shows the electrochemical impedance of Si-Sb-(ZnO)_*x*_ composite anodes and the fitting results before cycling. All the plots were composed of semicircles with a large radius of curvature and sloping lines. The semicircle in the high-frequency intercept represents the contact resistance without lithium insertion, charge transfer, and lithium-ion diffusion at the open voltage [26, 27]. The inset of Fig. 4d shows the impedance responses, which were then analyzed using an equivalent circuit that takes into account all possible contributions to the impedance of the test cell, using *R*_e_ as the electrolyte impedance, *Q*_cf_ and *R*_cf_ as the contact resistances, and *Q*_g_ as the diffusive resistance, and the representative fitting results are shown in Table 2. As can be seen, the appropriate addition of ZnO decreases aggregation in the immiscible Si-Sb system, promoting electrolyte infiltration while reducing the contact resistance of the electrode. However, since the electrical conductivity of ZnO is relatively lower than the alloy, an excess of ZnO addition would lead to the increase of electrode resistance instead.

**Table 2:** Impedance parameters associated to Si-Sb-(ZnO)_*x*_ anode materials at open circuit voltage

Sample	*R*_e_/Ω	*R*_cf_/Ω
Si-Sb	5.413	401.3
Si-Sb-(ZnO)_0.1_	3.635	380.1
Si-Sb-(ZnO)_0.3_	3.452	263.7
Si-Sb-(ZnO)_0.5_	4.332	275.2
Si-Sb-(ZnO)_0.7_	5.461	281.9
Si-Sb-(ZnO)_0.9_	5.993	299.9

**Fig. 5:**
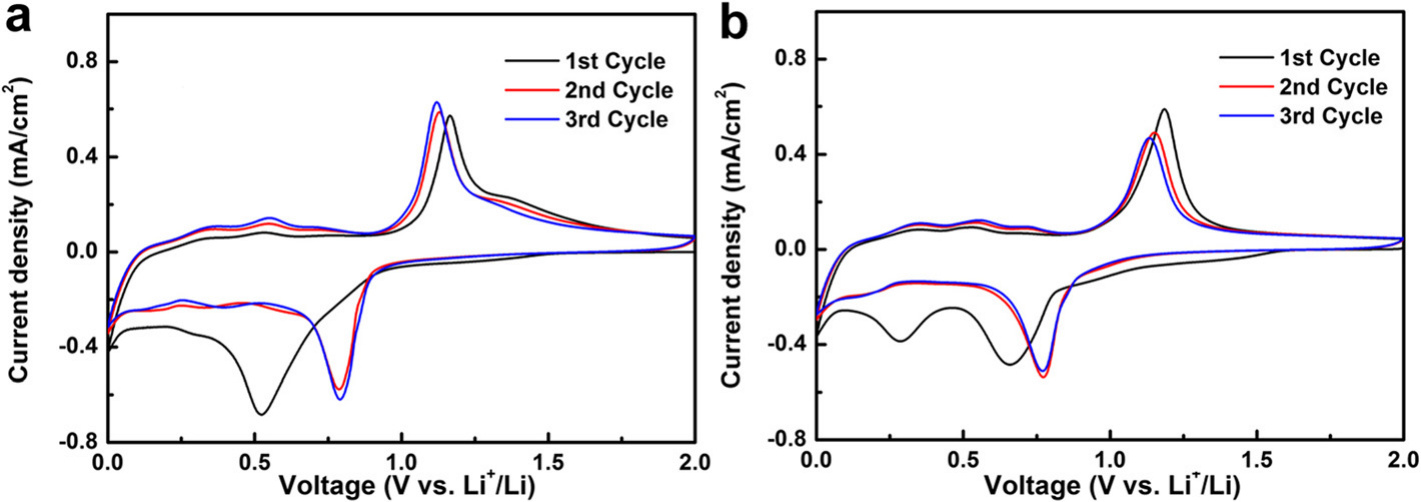
CV curves of Si-Sb (**a**), and Si-Sb-(ZnO)_0.3_ (**b**) composite materials at a scanning rate of 0.01 mV/s

Cyclic voltammograms of Si-Sb and Si-Sb-(ZnO)_0.3_ composite material were tested in the voltage range of 0–2.0 V (vs. Li/Li^+^) at a scanning rate of 0.01 mV/s. As shown in Fig. 5, there is almost no change of the curve for the 2nd and 3rd cycles, indicating a high reversibility, and the relatively large difference between the Si-Sb and Si-Sb-(ZnO)_0.3_ is due to the lithium intercalation reactions with ZnO. For the cathodic scan, a low reduction peak is observed in the first cycle at 0.24 V, but disappears in subsequent cycles, corresponding to the reduction of the oxides, the decomposition of the electrolyte, and the formation of SEI film. A strong reduction peak appeared at about 0.8 V and persisted through subsequent cycles, which corresponded to Sb in the Si-Sb alloy reacting with Li to form Li_3_Sb. Afterwards, a series of peaks appeared after 0.6 V, corresponding to the reaction of ZnO with Li to form LiZn and the lithiation of Si to form a series of Li_*x*_Si alloys, etc. In the anodic scan, a series of weak peaks appeared at 0.3–0.5 V, corresponding to the de-lithiation of Li_*x*_Si, and the strong peak at 1.1 V was attributed to the formation of Si-Sb alloys.

**Fig. 6:**
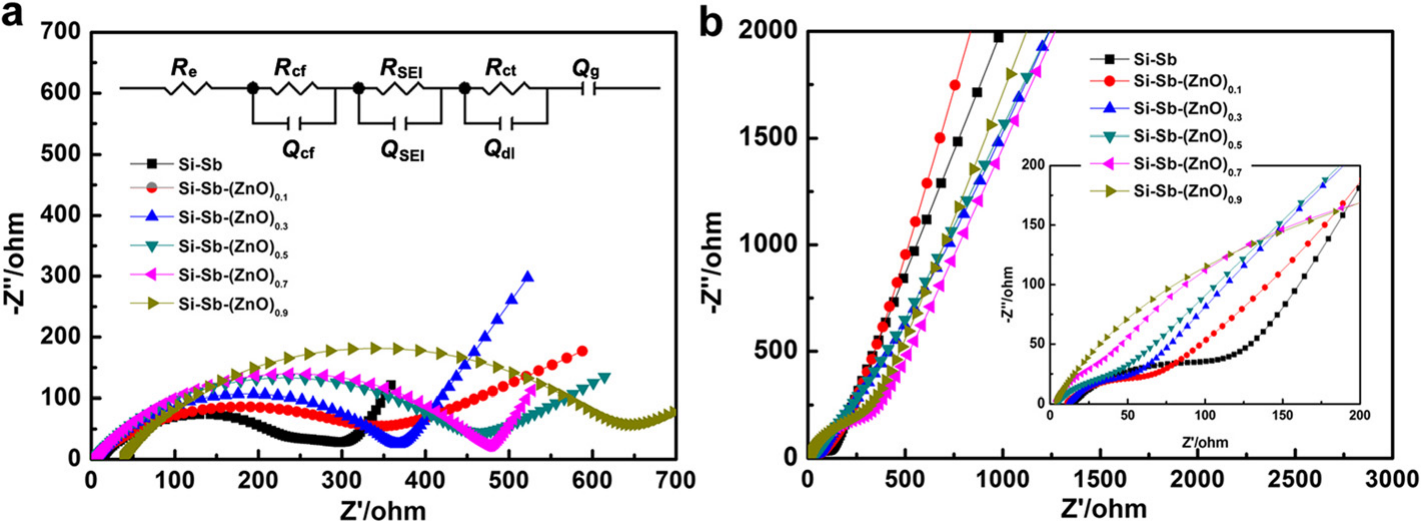
EIS of Si-Sb-(ZnO)_*x*_ anode materials after first lithium insertion (a), and extraction process (**b**). The inset of **a** is the associated equivalent circuit

Figure 6 compares the impedance response of Si-Sb-(ZnO)_*x*_ and the representative fitting results after the first lithium insertion/extraction cycles. The radius of the semicircles in the low-frequency are observed to increase after the lithium insertion process which is due to the generated Li_*x*_Sb, Li_*x*_Si, and Li_*x*_Zn packed on the active particles and blocked the transport channels for lithium and electrons, resulting in an increase in the impedance. During de-lithiation process, the composites decomposed, recovering the porous electrode structure and unblocking the channels; therefore, the internal impedance decreased. It is noted that Si-Sb-(ZnO)_0.3_ shows the smallest radius of the semicircle in the low-frequency, indicating the highest electrical conductivity among the composites, which is consistent to the above results.

## Conclusions

Si-Sb-(ZnO)_*x*_ (*x* = 0, 0.1, 0.3, 0.5, 0.7, 0.9) composite anode materials were prepared by a chemical reduction-mechanical alloying method. The materials exhibited superior capacity and good cycling stability for lithium ion-batteries. When the molar ratio of ZnO was 0.3, the initial specific charge capacity and specific discharge capacity of the synthesized Si-Sb-(ZnO)_0.3_ composite anode material were 870.3 and 1301.5 mAh/g, respectively. An appropriate amount of ZnO was able to effectively relieve the agglomeration of the Si-Sb alloy particles, not only greatly shortening the lithium-ion diffusion distance, but also increasing the space available for volume expansions, resulting in greatly improved cycling stability for the Si-Sb-(ZnO)_*x*_ composite anode materials. This study gives a rational direction for tailoring the components and structures of composite anode materials to improve the performance of lithium-ion batteries.

## Additional File

Additional file 1: Figure S1. Cycling performance of ZnO, Si-Sb, Si-Sb-(ZnO)_0.3_ anode materials.
