# Supplementary material for: Synthesis of Si-Sb-ZnO Composites as High-Performance Anodes for Lithium-ion Batteries
Source: Nanoscale Res Lett. 2015 Oct 23;10:414. doi: 10.1186/s11671-015-1128-4 (PMC4615995; doi:10.1186/s11671-015-1128-4)
Supplement: Additional file 1: Figure S1. — Cycling performance of ZnO, Si-Sb, Si-Sb-(ZnO)0.3 anode materials. [file 11671_2015_1128_MOESM1_ESM.docx]

**Synthesis of Si-Sb-ZnO Composites as High Performance Anodes for Lithium-ion Batteries**

Yongliang Li, Liang Huang, Peixin Zhang*, Xiangzhong Ren, Libo Deng

(School of Chemistry and Environmental Engineering, Shenzhen University, Shenzhen, Guangdong, P.R. China, 518060)

*Corresponding author: Peixin Zhang, Tel & Fax: 86-755-26733136, Email: pxzhang@szu.edu.cn





**Fig. S1** Cycling performance of ZnO, Si-Sb, Si-Sb-(ZnO)_0.3_ anode materials.
